# Notch Signaling Activation Enhances Human Adipose-Derived Stem Cell Retinal Differentiation

**DOI:** 10.1155/2018/9201374

**Published:** 2018-10-16

**Authors:** Yuqiang Huang, Tsz Kin Ng, Chong-Bo Chen, Bing Huang, Jiajian Liang, Chi Pui Pang, Mingzhi Zhang

**Affiliations:** ^1^Joint Shantou International Eye Center of Shantou University and the Chinese University of Hong Kong, Shantou, Guangdong, China; ^2^Shantou University Medical College, Shantou, Guandong, China; ^3^Department of Ophthalmology and Visual Sciences, The Chinese University of Hong Kong, Hong Kong

## Abstract

Retinal disease treatment by stem cell-based replacement relies on stem cell differentiation into retinal cells. We previously demonstrated that human periodontal ligament-derived stem cells can be directed into retinal lineage upon induction. Here, we report the transdifferentiation potential of human adipose-derived stem cells (ASCs) into retinal lineage and its enhancement by Notch signaling modulation. Human ASCs, isolated from abdominal fat, expressed mesenchymal but not hematopoietic stem cell markers, and they can differentiate into adipocytes, chondrocytes, and osteoblasts *in vitro*. Upon noggin/Dkk-1/IGF-1 induction, the treated ASCs showed elevated expression of retinal progenitor, retinal ganglion, and photoreceptor cell markers as well as the glutamate-evoked calcium response, which was not observed in the noninduced cells. Compared to the regular induction treatment, Notch signaling activation by JAG1 enhanced the expression of retinal progenitor and precursor markers without affecting the glutamate-evoked calcium response. In contrast, Notch signaling inhibition by DAPT showed more retinal ganglion cells, but delayed the response to glutamate stimulation. In summary, our results revealed that human ASCs possess a retinal transdifferentiation potential upon noggin/Dkk-1/IGF-1 induction, which can further be enhanced by Notch signaling activation.

## 1. Introduction

Retinal diseases, including age-related macular degeneration, glaucoma, diabetic retinopathy, and retinitis pigmentosa, are the leading causes of irreversible blindness and visual impairment, affecting more than 300 million people worldwide. The human retina has limited intrinsic regenerative capability, and current treatment regimens are not sufficient for functional repair of the diseased retinal cells. Stem cell-based replacement, which relies on the differentiation of stem cells into retinal cells, could be a potential strategy for retinal disease treatment [1, 2].

Retinal progenitor cells (RPCs), embryonic stem cells (ESCs), and induced pluripotent stem cells (iPSCs) have been intensively studied for retinal cell synthesis [3, 4]. Yet, the studies on human adult stem cells, which can be conveniently obtained for autologous transplantation, for retinal differentiation are limited. We have established protocols to direct human periodontal ligament-derived stem cells (PDLSCs) into retinal lineage by inhibiting the bone morphogenetic protein (BMP) and Wnt signaling pathways with supplementation of insulin-like growth factor 1 (IGF-1) [5, 6]. We postulate that our retinal differentiation strategy can be generally applied to different types of adult stem cells.

Adipose tissue, derived from the mesoderm, can safely be obtained by liposuction and contains an abundance of adipose-derived stem cells (ASCs). Human ASCs possess a multilineage differentiation potential with a low risk of inducing autoimmunity and teratoma formation [7]. They can express retinal and retinal pigment epithelial (RPE) cell markers when cultured in RPE-conditioned medium [8, 9]. Moreover, ASCs can also be induced to retinal cells *in vitro* with ectopic expression of paired box protein-6 (*PAX6*) gene in a fibronectin-supplemented medium [10]. These suggested that human ASCs should possess a transdifferentiation potential towards retinal lineage.

Apart from the BMP, Wnt, and IGF-1 signaling pathways, the Notch signaling pathway is also involved in different developmental processes, including retinogenesis and neurogenesis, controlling cell fate decisions [11, 12]. Previous studies showed that Notch signaling activation promotes RPC proliferation and prevents RPC differentiation into retinal cells [12]. Moreover, Notch-1 signaling is involved in the regulation of photoreceptor development at early and late stages of retinal development [13]. Inactivation or removal of Notch-1 from RPCs increases photoreceptor precursor production in mice [14]. Similarly, inhibition of Notch promotes the generation of photoreceptor precursors from human iPS-derived RPCs [15]. In addition, reducing Notch expression increases retinal ganglion cell (RGC) genesis [16]. Nevertheless, Notch-1^+^ progenitor cells express both Atoh7 and Otx2, suggesting a role of Notch1 signaling in the differentiation of RGCs and photoreceptors [17]. However, the role of Notch signaling in the retinal differentiation of human adult stem cells remains elusive. Herein, we hypothesize that Notch signaling modulation can enhance the transdifferentiation of human adult stem cells towards retinal lineage. This study determined the transdifferentiation potential of human ASCs into retinal lineage by noggin/Dkk-1/IGF-1 induction. We also delineated the role of Notch signaling in human ASCs' retinal differentiation.

## 2. Materials and Methods

### 2.1. Isolation and Maintenance of Human Adipose-Derived Stem Cells

Human ASCs were obtained from liposuction of abdominal fat in 3 healthy subjects after informed written consent. This study was approved by the Ethnic Committee of Human Study in the Joint Shantou International Eye Center of Shantou University and the Chinese University of Hong Kong. Briefly, after specimen collection from donors, the excess muscle tissues, nonfat tissues, and blood trace were first removed from the adipose tissue, which was then minced and centrifuged. The floating tissue was separated and digested with type I collagenase at 37°C for 2 hours. After enzyme blockage, the digested tissue was filtered through a 100 *μ*m nylon mesh and centrifuged. Human ASCs were maintained in ADSC-BM medium (Lonza) supplemented with 10% fetal bovine serum (FBS; Gibco), 1% glutamine, and 0.1% antibiotics GA-1000. The medium was replenished every 3 days, and the cells were passaged when reaching 60–70% confluence. Human ASCs at passages 3–5 were used in this study.

### 2.2. Stem Cell Marker Expression in Human Adipose-Derived Stem Cells

The isolated human ASCs were characterized by the expressions of mesenchymal stem cell (MSC: CD44, CD73, CD90, and CD105) and hematopoietic stem cell (HSC: CD14, CD34, and CD45) markers using flow cytometry according to the manufacturer's protocol (BD Biosciences). Briefly, human ASCs were dissociated from culture dishes by 0.05% trypsin/EDTA, washed twice with cold stain buffer (BD Biosciences), and resuspended in cold stain buffer at a concentration of 2 × 10^7^ cells/mL. The cell suspension was incubated with respective antibodies (Supplementary Table 1) or isotype controls for 30 min on ice covered with aluminum foil to avoid light. After antibody probing, the cells were washed twice with stain buffer and analyzed by the Accuri C6 Flow Cytometer (BD Biosciences) with at least 10,000 events. The percentage of positive cells was calculated with respect to the isotype control cells.

### 2.3. Mesenchymal Lineage Differentiation of Human Adipose-Derived Stem Cells

Adipogenesis differentiation of human ASCs was induced by the adipocyte differentiation basal medium supplemented with 10% adipocyte supplement (StemPro, Gibco) for 14 days. The treated cells were fixed with 4% (*w*/*v*) paraformaldehyde (pH 7.4; Sigma-Aldrich) and stained with Oil Red O reagent for 10 min at ambient temperature.

Osteogenesis differentiation of human ASCs was induced by the osteocyte/chondrocyte differentiation basal medium containing with 10% osteocyte supplement (StemPro, Gibco) for 14 days. For alkaline phosphatase activity detection, the cells were fixed in ice-cold acetone and incubated with NBT/BCIP solution for 20 min. For calcium deposition analysis, the cells were fixed in 4% paraformaldehyde and stained with Alizarin Red S (Sigma-Aldrich) for 2-3 min.

Chondrogenesis differentiation of human ASCs was induced by the osteocyte/chondrocyte differentiation basal medium with 10% chondrocyte supplement (StemPro, Gibco) on a Matrigel coating for 14 days. The resulting chondrogenic pellets were fixed in 4% (*w*/*v*) paraformaldehyde (pH 7.4), dehydrated, and imbedded in OCT. The imbedded cell pellets were cryosectioned and stained with Alcian blue (Sigma-Aldrich) staining for 30 min.

The negative control was the ASCs without the addition of the adipocyte, osteocyte, or chondrocyte supplements.

### 2.4. Retinal Induction of Human Adipose-Derived Stem Cells

Retinal induction treatment was divided into the negative control group and 3 induction groups. For the negative control group, the cells were treated with 10% FBS-supplemented 1 : 1 Dulbecco's Modified Eagle Medium: nutrient mixture F-12 (DMEM/F12; Gibco) medium in the ultralow attachment culture dish (Corning) for 3 days and then in the Matrigel-coated (Gibco) culture dish for 21 days. For the retinal induction groups, a three-stage noggin/Dkk-1/IGF-1 induction approach was adopted. Firstly, human ASCs (5 × 10^5^ cells) were aggregated in the ultralow attachment culture dish with the induction medium 1 (IM1; DMEM/F12 medium supplemented with 10% knockout serum replacement (Gibco), 1x B27 supplement (Gibco), 1 ng/mL noggin (PeproTech), 1 ng/mL dickkopf-related protein 1 (Dkk-1; PeproTech), and 5 ng/mL IGF-1 (PeproTech)) for 3 days. The aggregated cells were then transferred to the Matrigel-coated culture dish and treated with the induction medium 2 (IM2; DMEM/F12 medium supplemented with 1x B27 supplement, 1x N2 supplement (Gibco), 10 ng/mL noggin, 10 ng/mL Dkk-1, 10 ng/mL IGF-1, and 5 ng/mL basic fibroblast growth factor (bFGF; PeproTech)) for 7 days. Afterwards, the culture medium was replaced by the induction medium 3 (IM3; DMEM/F12 medium supplemented with 1x B27 supplement, 1x insulin-transferrin-selenium (ITS; Gibco), 10 ng/mL noggin, 10 ng/mL Dkk-1, 10 ng/mL IGF-1, and 5 ng/mL bFGF) and further treated for 2 weeks. To delineate the role of Notch signaling regulation in human ASCs' retinal differentiation, JAG1 (20 *μ*M; Notch signaling activator) and *γ*-secretase inhibitor (DAPT, 10 *μ*M; Notch signal inhibitor) were supplemented into IM3 and treated for 2 weeks. Medium was replenished every 3 days. The treated cells were collected at Days 0, 10, 17, and 24 for further analyses.

### 2.5. RNA and Protein Isolation

After medium aspiration, the treated cells were directly lysed with the addition of 350 *μ*L Buffer RP1 supplemented with 3.5 *μ*L of *β*-mercaptoethanol (Sangon Biotech). RNA and protein were extracted simultaneously according to the manufacturer's protocol (NucleoSpin RNA/Protein, Macherey-Nagel). RNA was eluted by the RNase-free water, whereas the protein pellet was dissolved in protein solving buffer containing TCEP. The extracted RNA and protein samples were stored at −80°C before respective gene expression and immunoblotting analyses.

### 2.6. Gene Expression Analysis

Total RNA concentration was measured by a NanoDrop ND-1000 spectrophotometer (Thermo Fisher Scientific). Total RNA (1 *μ*g) was reverse transcribed into complementary DNA (cDNA) using the PrimeScript RT reagent Kit (TaKaRa) in a thermal cycler (S1000 Thermal Cycler, Bio-Rad). The expression of RPC (*PAX6* and *NES*), RGC (*ATOH7* and *TUBB3*), photoreceptor (*CRX*, *NRL*, *RHO*, and *RCVRN*), and Notch signaling (*NOTCH1* and *HES1*) markers was evaluated by the QuantiNova SYBR Green PCR Kit (Qiagen) with specific primers (Supplementary Table 2) in a LightCycler480 (Roche). A housekeeping gene (*ACTB*) was used for normalization.

### 2.7. Immunofluorescence Analysis

The treated human ASCs on the Matrigel-coated coverslips were fixed with the freshly prepared 4% (*w*/*v*) paraformaldehyde in PBS (pH 7.4) for 15 min at ambient temperature. The fixed cells were rinsed three times in PBS, and the remaining free aldehyde was quenched by 50 mM ice-cold NH_4_Cl solution. After washing, the cells were permeated and blocked by PBS supplemented with 0.15% (*w*/*v*) saponin, 1% (*w*/*v*) bovine serum albumin (BSA), 1% (*v*/*v*) normal goat serum (NGS), 0.01% Triton X-100, and 0.01% Tween-20 for 10 min. After blocking, the cells were incubated with primary antibodies (Supplementary Table 1; diluted in PBS containing 0.425% saponin, 1% BSA, 1% NGS, 0.0015% Triton X-100, and 0.0015% Tween-20) for 18 hours at 4°C. The cells were then incubated with the respective fluorophore-conjugated secondary antibody and 0.1% (*v*/*v*) DAPI (diluted in PBS containing 1% BSA and 1% NGS) for 1 hour at ambient temperature. The cells were mounted, and the fluorescence signals were visualized under a confocal microscope (TCS SP5, Leica). Nine fields were imaged and means of the 3 independent human ASC samples from different treatment groups were compared.

### 2.8. Immunoblotting Analysis

Total protein was quantified by the BCA protein assay. An equal amount of total protein (20 *μ*g) was heat denatured, resolved by 12.5% SDS-polyacrylamide gel electrophoresis, and transferred to the PVDF membrane. After blocking with 5% nonfat milk, the blot was probed with primary antibodies for RPC (PAX6) and photoreceptor (RHO) markers (Supplementary Table 1) for 18 hours at 4°C, followed by the respective horseradish peroxidase- (HRP-) conjugated secondary antibodies. The signals were detected by enhanced chemiluminescence (Santa Cruz Biotechnology, Inc.) and imaged by the ChemiDoC XRS^+^ system (Bio-Rad). GAPDH was used as a housekeeping protein for normalization.

### 2.9. Glutamate-Evoked Calcium Response

Spontaneous intracellular calcium transient was evaluated using fluo-4-acetoxymethyl ester (Fluo-4 AM; Invitrogen). Briefly, the treated or control human ASCs at Day 24 were incubated in Hanks' balanced salt solution (HBSS, Ca^2+^/Mg^2+^-free; Gibco) containing 5 *μ*M Fluo-4 AM and 0.1% pluronic F-127 (Invitrogen) for 30 min at room temperature. After washing with HBSS, 1 mM glutamate (Sigma-Aldrich) was added, and fluorescence images were immediately captured using a confocal microscope (Leica TCS SP5), with the excitation wavelength at 495 nm and emission wavelength at 515 nm, every 1 second in a total of 5 min under a 20x objective lens. Fluorescence intensity at specific time intervals was measured on a total of 50 cells in triplicate experiments (at least 10 cells in each sample). The cellular change of fluorescence (*F*; %Δ*F*/*F*_baseline_) of each region was calculated as (*F*_treated_ − *F*_baseline_)/*F*_baseline_ × 100%. The Ca^2+^ fluorescence ratio was converted into Ca^2+^ concentration using the equation [Ca^2+^]_*i*_ = *KR*/{*K*/([Ca^2+^]_rest_ + 1) − *R*}, where *K* is the dissociation constant of Fluo-4 AM (400 nM), *R* is the fluorescence ratio (Δ*F*/*F*_baseline_), and [Ca^2+^]_rest_ is the resting Ca^2+^ concentration (100 nM in neuronal cells).

### 2.10. Statistical Analysis

The data were presented as mean ± standard deviation (SD) from the 3 independent cell lines. All statistical analyses were performed using a commercially available software (SPSS, version 20.0; SPSS Inc., Chicago, IL). Independent *t*-test and one-way analysis of variance (ANOVA) with post hoc Tukey's test were used to compare the means among the treatment groups. Significance was defined as *p* < 0.05.

## 3. Results

### 3.1. Characterization of Human Adipose Tissue-Derived Stem Cells

**Figure 1:**
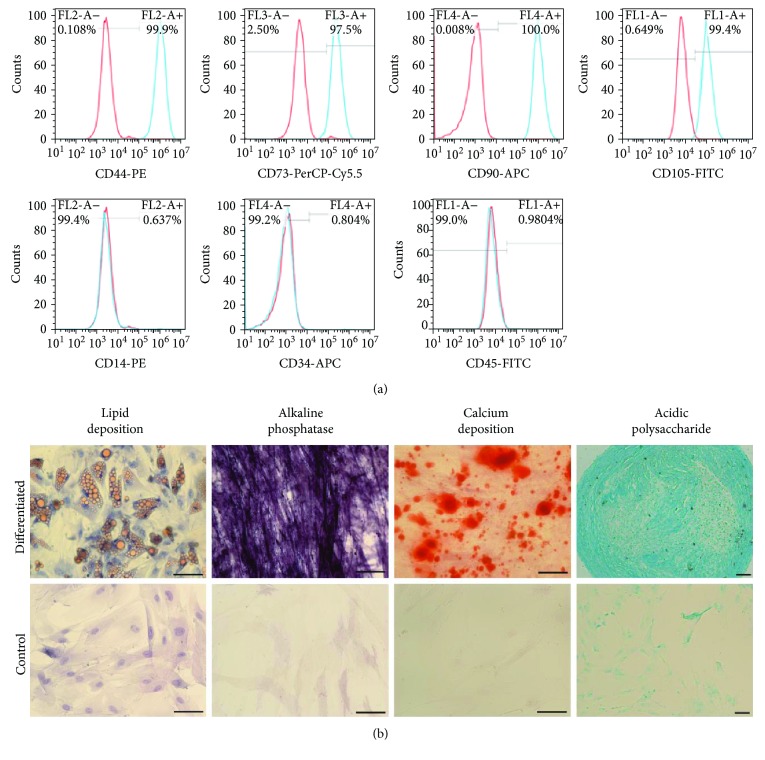
Characterization of human adipose-derived stem cells. (a) Human ASCs were characterized by flow cytometry based on the expression of mesenchymal stem cell (CD44, CD73, CD90, and CD105) and hematopoietic stem cell (CD14, CD34, and CD45) markers. The red histograms refer to the isotype controls, and the blue histograms refer to the human ASC samples for the test. (b) ASCs were differentiated into adipocytes, osteoblasts, and chondrocytes upon induction. Adipogenesis differentiation was evaluated by Oil Red O staining (red) and hematoxylin counterstain (blue), and the yellowish lipid depositions were observed in the differentiated cells. For the osteogenesis differentiation, the NBT/BCIP and Alizarin Red S staining demonstrated that the differentiated cells possess strong alkaline phosphatase activity (blue) and reddish calcium deposition, respectively. Chondrogenesis differentiation was evaluated by Alcian blue staining, and the production of acidic polysaccharide (light blue) can be observed in the differentiated cells. Scale bar: 100 *μ*m.

Human ASCs were first characterized by the expression of MSC (CD44, CD73, CD90, and CD105) and HSC (CD14, CD34, and CD45) markers. Flow cytometry analysis showed that CD44 expression was found in 99.10 ± 1.01%, CD73 in 97.07 ± 2.56%, CD90 in 99.90 ± 0.24%, and CD105 in 99.45 ± 0.39% of human ASCs (Figure 1(a)). In contrast, 0.17 ± 0.23% of human ASCs were positive for CD14, 1.42 ± 1.40% for CD34, and 2.53 ± 1.41% for CD45. To determine the differentiation ability of the isolated ASCs, adipogenesis, osteogenesis, and chondrogenesis of human ASCs were evaluated. More than 90% of human ASCs could differentiate into adipocytes with yellowish lipid deposition (Figure 1(b)). Moreover, human ASCs could also differentiate into osteoblasts, indicated by intensive alkaline phosphatase activity and the reddish calcium deposition, as well as chondrocytes, indicated by the light blue staining of acidic polysaccharide. Our results indicated that human ASCs isolated from abdominal fat were predominantly functional MSCs.

### 3.2. Retinal Differentiation of Human Adipose Tissue-Derived Stem Cells

**Figure 2:**
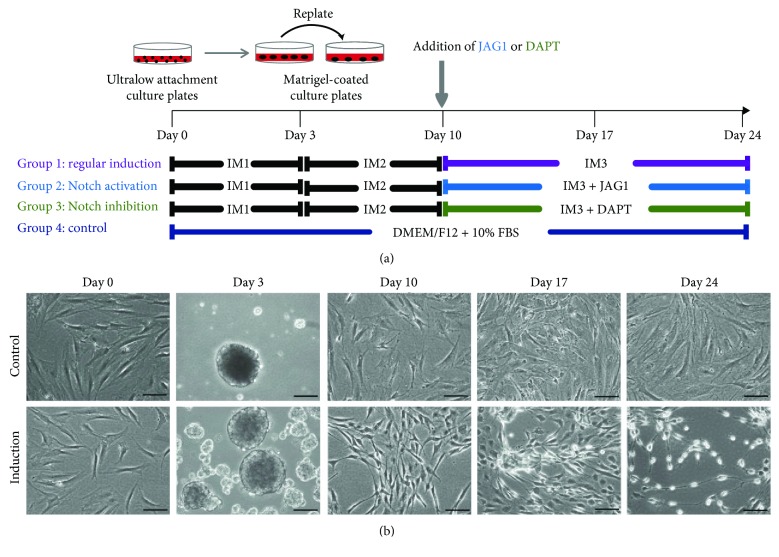
Induction treatment of human adipose-derived stem cells into the retinal lineage. (a) A schematic diagram of the induction treatment of human ASCs into the retinal lineage. Human ASCs were first aggregated in IM1 for 3 days and cultured on Matrigel-coated plates for 7 days in IM2 and 14 days in IM3. Notch signaling activation and inhibition were mediated by JAG1 and DAPT, respectively. The control cells were cultured with DMEM/F12 + 10% FBS for 24 days. (b) The morphology of human ASCs after retinal induction treatment at different time points. Upper: noninduction group (control). Lower: regular induction group. The cells in the regular induction group at Day 24 exhibited a neural-network-like morphology with condensed cell bodies, while the cells in the control group remained in a fibroblast-like shape. Scale bar: 100 *μ*m.

Human ASCs were treated with retinal induction medium for 24 days (Figure 2(a)). A sphere-like structure was observed on Day 3 under the low adherent culture. On the Matrigel-coated surface, the aggregated ASCs migrated out from the rosette and exhibited neural-network-like morphology with condensed cell bodies (Figure 2(b)). In contrast, human ASCs in the negative control group remained in the fibroblast-like shape and proliferated continuously.

**Figure 3:**
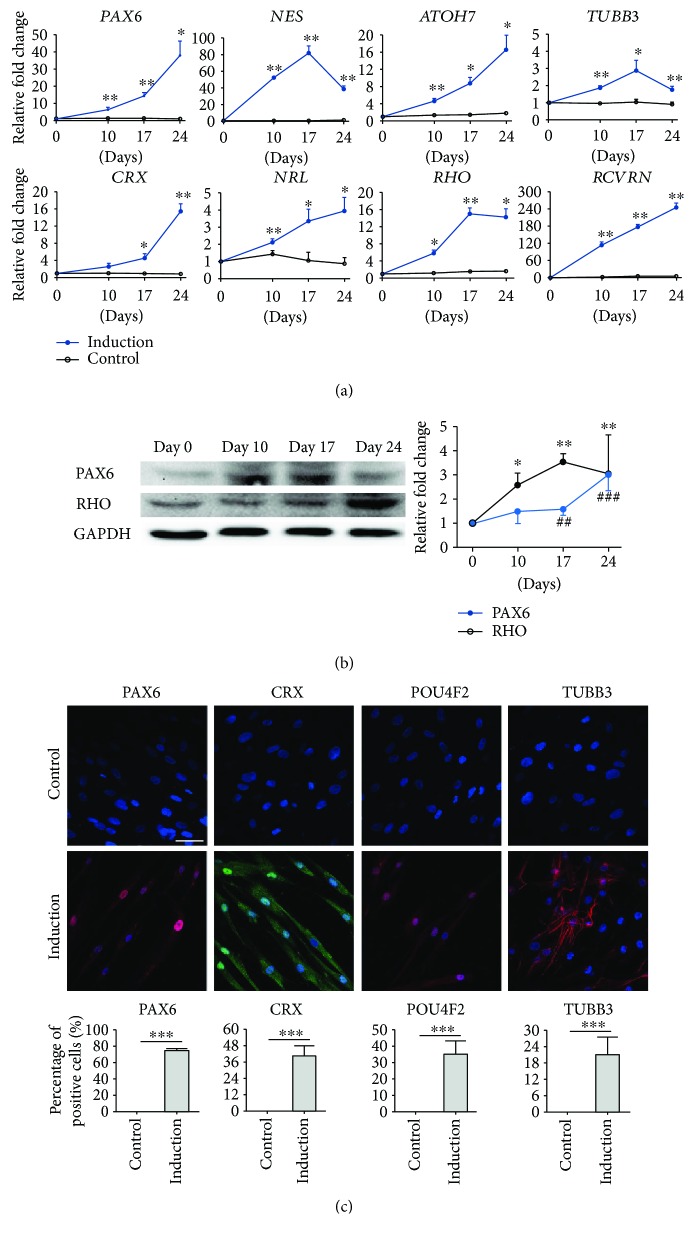
Retinal lineage marker expression in human adipose-derived stem cells after retinal induction treatment. (a) Gene expression analysis of retinal lineage markers: retinal progenitor cells (*PAX6* and *NES*), photoreceptor cells (*CRX*, *NRL*, *RHO*, and *RCVRN*), and retinal ganglion cells (*ATOH7* and *TUBB3*) (^∗^ *p* < 0.05 and ^∗∗^ *p* < 0.01 compared to the control group). (b) Protein expression analysis of the retinal progenitor cell (PAX6) and photoreceptor marker (RHO) by immunoblotting analysis. GAPDH was used as the housekeeping protein for normalization (^∗^ *p* < 0.05 and ^∗∗^ *p* < 0.01, compared to Day 0 in PAX6; ^##^ *p* < 0.01 and ^###^ *p* < 0.001, compared to Day 0 in RHO). (c) Immunofluorescence analysis of retinal lineage markers: retinal progenitor cell (PAX6; nucleus), photoreceptor precursor (CRX; nucleus), and retinal ganglion cell (POU4F2; nucleus and TUBB3). DAPI was used as the nucleus counterstain. Scale bar: 50 *μ*m. The bar chats represent the percentage of expression of markers in the control and induction groups (^∗∗∗^ *p* < 0.001).

To verify the retinal differentiation capacity of human ASCs, the expression of retinal lineage markers was examined at Days 0, 10, 17, and 24 after induction. Gene expression analysis showed that the expression of RPC (*PAX6* and *NES*), photoreceptor (*CRX*, *NRL*, *RHO*, and *RCVRN*), and RGC (*ATOH7* and *TUBB3*) marker genes time-dependently increased along the retinal induction treatment period (Figure 3(a)). Sybr green PCR analysis demonstrated that, at Day 24, *PAX6* and *NES* genes were increased in the treated human ASCs by 38.03 ± 8.41-fold (*p* < 0.05) and 38.41 ± 4.12-fold (*p* < 0.01), respectively, compared to the expression at Day 0. Similarly, *CRX*, *NRL*, *RHO*, and *RCVRN* genes were increased by 15.46 ± 1.75-fold (*p* < 0.05), 3.94 ± 0.81-fold (*p* < 0.05), 14.25 ± 1.96-fold (*p* < 0.05), and 245.90 ± 15.46-fold (*p* < 0.05), respectively. Meanwhile, the expression of *ATOH7* and *TUBB3* genes in the retinal-induced ASCs at Day 24 were increased by 16.65 ± 3.35-fold (*p* < 0.05) and 1.75 ± 0.19-fold (*p* < 0.05), respectively. In contrast, all retinal marker genes showed no significant change in the negative control group throughout the treatment period.

Coherent to the gene expression results, the expressions of PAX6 and RHO protein were time-dependently upregulated in retinal-induced ASCs, with 3.06 ± 1.31-fold (*p* < 0.01) and 3.00 ± 0.53-fold increase (*p* < 0.001, Figure 3(b)) at Day 24, respectively, compared to the expressions at Day 0. Moreover, immunofluorescence analysis showed that the retinal-induced ASCs expressed PAX6 (74.74 ± 2.42%, *p* < 0.001), CRX (40.72 ± 7.01%, *p* < 0.001), POU4F2 (35.10 ± 7.86%, *p* < 0.001), and TUBB3 (21.25 ± 6.18%, *p* < 0.001; Figure 3(c)). In contrast, retinal marker expression was not observed in the ASCs of the negative control group.

**Figure 4:**
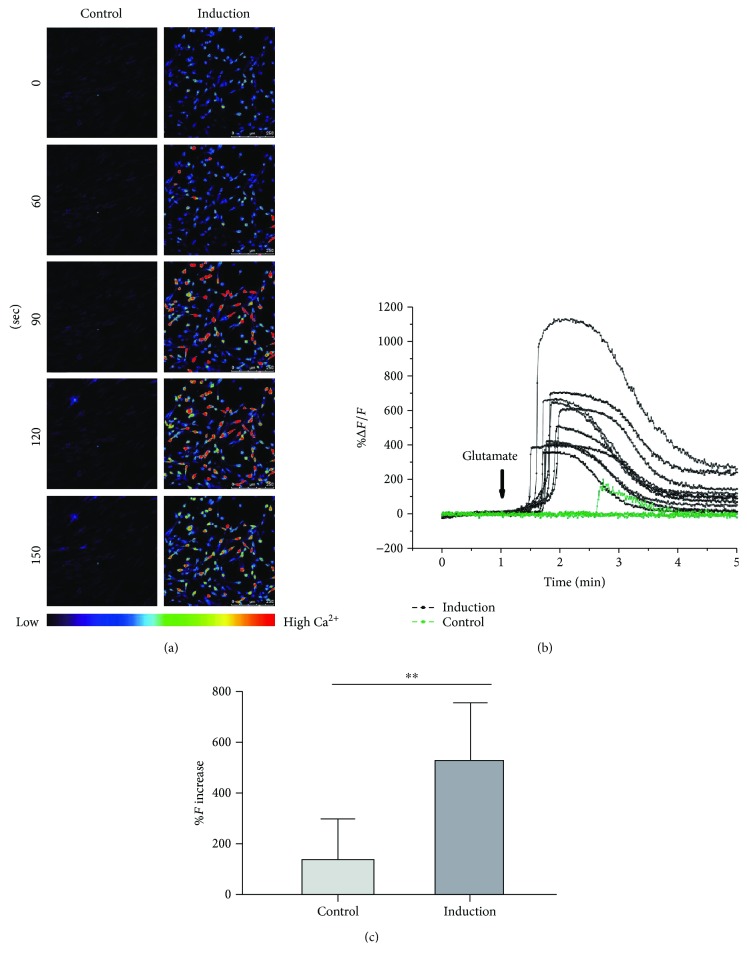
Glutamate-evoked calcium response in retinal-induced human adipose-derived stem cells. Human ASCs were treated under retinal induction treatment for 24 days. (a) Heat map images show the Fluo-4 AM fluorescence in the control and induction groups after 1 mM glutamate treatment for 0, 60, 90, 120, and 150 seconds. Scale bar: 250 *μ*m. (b) Representative traces for spontaneous calcium transient profiles of retinal-induced ASCs (black) and control ASCs (green) are shown. (c) Peak calcium response (mean ± standard deviation) in the induction and control ASCs was compared to baseline levels (^∗∗^ *p* < 0.01).

The function of the retinal-induced ASCs was evaluated by the glutamate-evoked calcium response. A robust increase in fluorescence intensities was observed in the retinal-induced ASCs after glutamate stimulation, but only a very weak signal was observed in the negative control group (Figure 4, Supplemental Video 1 and Supplemental Video 2). The fluorescence intensity changes (%Δ*F*/*F*_0_) observed in the retinal-induced ASCs were 525.18 ± 228.80%, compared to 133.79 ± 159.82% in the negative control group (*p* < 0.01).

To confirm the specificity of retinal transdifferentiation, the retinal-induced ASCs were evaluated with the staining for adipogenesis and osteogenesis, and negative staining of lipid deposition, alkaline phosphatase, and calcium deposition was observed in the retinal-induced ASCs (Supplementary Figure 1). Collectively, our results indicated that human ASCs, upon noggin/Dkk-1/IGF-1 treatment, successfully and specifically transdifferentiated into functional retinal cells.

### 3.3. Notch Signaling Regulation in Retinal Differentiation of Human Adipose Tissue-Derived Stem Cells

**Figure 5:**
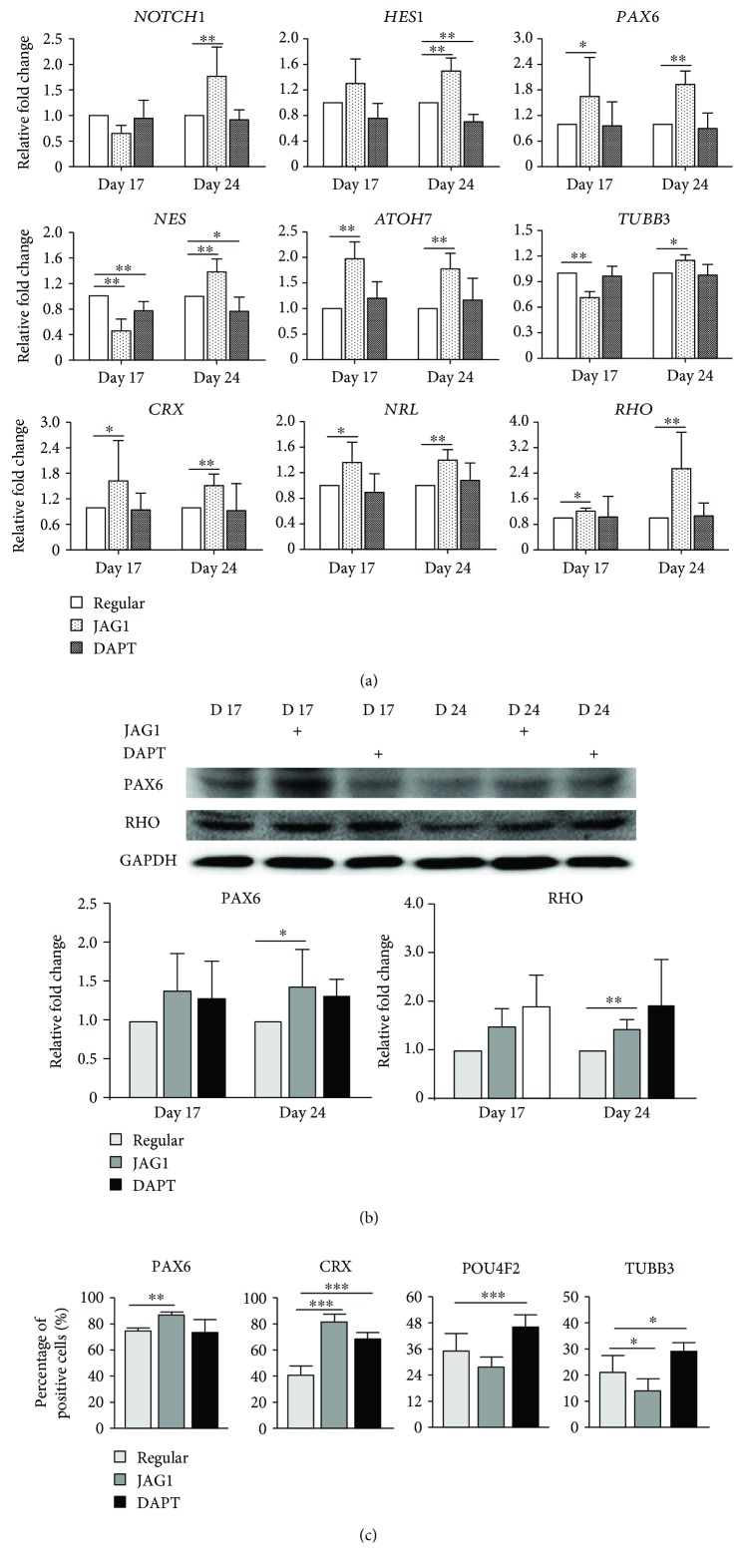
Retinal lineage marker expression in retinal-induced human adipose-derived stem cells upon Notch signaling modulation. (a) Gene expression analysis for markers upon Notch signaling modulation: Notch signaling effector *HES1* and Notch receptor *NOTCH1*; retinal progenitor cell markers *PAX6* and *NES*; photoreceptor cell markers *CRX*, *NRL*, and *RHO*; retinal ganglion cell markers *ATOH7* and *TUBB3*. Relative fold change was compared to the regular induction group at Days 17 and 24 (^∗^ *p* < 0.05 and ^∗∗^ *p* < 0.01). (b) Immunoblotting analysis for the retinal progenitor marker (PAX6) and photoreceptor marker (RHO). GAPDH was used as the housekeeping gene for normalization (^∗^ *p* < 0.05 and ^∗∗^ *p* < 0.01). (c) Immunofluorescence analysis of *PAX6*, CRX, POU4F2, and TUBB3 expressions in retinal-induced ASCs upon Notch signaling modulation treatments (^∗^ *p* < 0.05, ^∗∗^ *p* < 0.01, and ^∗∗∗^ *p* < 0.001).

To delineate the regulation of Notch signaling in the retinal differentiation of human ASCs, JAG1 (Notch signaling activator) and DAPT (Notch signaling inhibitor) were applied to the retinal induction medium (Figure 2(a)). The efficacy of Notch signaling modulators on human ASCs was confirmed by the expression of the Notch signaling downstream effector (*HES1* gene). In the JAG1 treatment group, the *HES1* gene was significantly upregulated by 1.50-fold at Day 24 (*p* < 0.01; Figure 5(a)), compared to the regular induction group, while, in the DAPT treatment group, the *HES1* gene was significantly downregulated by 1.43-fold at Day 24 (*p* < 0.01). Notably, the expression of the Notch signaling receptor (*NOTCH1* gene) was also upregulated in the JAG1 treatment group by 1.76-fold (*p* < 0.01), but not changed in the DAPT treatment group.

For the gene expression analysis on RPC markers, the expression of the *PAX6* gene was upregulated in the JAG1 treatment group by 1.93 ± 0.31-fold at Day 24 (*p* < 0.001), compared to the regular induction group. Similarly, the expression of the *NES* gene was also increased in the JAG1 treatment group by 1.37 ± 0.21-fold at Day 24 (*p* < 0.05). In contrast, the expression of the *NES* gene was downregulated in the DAPT treatment group by 1.32 ± 0.22-fold (*p* < 0.05). For the photoreceptor markers, the expressions of *CRX*, *NRL*, and *RHO* genes were upregulated by 1.51 ± 0.28-fold (*p* < 0.01), 1.40 ± 0.17-fold (*p* < 0.01), and 2.55 ± 1.15-fold (*p* < 0.001), respectively, in the JAG1 treatment group at Day 24, compared to those in the regular induction group. For the RGC markers, the expressions of the *ATOH7* and *TUBB3* genes were increased in the JAG1 treatment group by 1.78 ± 0.31-fold (*p* < 0.01) and 1.15 ± 0.06-fold (*p* < 0.05), respectively, at Day 24, compared to the regular induction group. Human ASCs in the DAPT treatment group showed a similar retinal marker gene expression to that in the regular induction treatment group.

Coherent to the gene expression results, the immunoblotting analysis showed that the expression of the PAX6 protein was significantly upregulated in the JAG1 treatment group by 1.45 ± 0.33-fold at Day 24 (*p* < 0.05), compared to the regular induction group (Figure 5(b)). Similarly, the expression of the RHO protein was also upregulated in the JAG1 treatment group by 1.47 ± 0.17-fold (*p* < 0.01) at Day 24, compared to the regular induction group. Furthermore, the percentage of PAX6 nucleus-positive cells in the JAG1 treatment group (87.06 ± 2.69%, *p* < 0.01) was higher than that in the regular induction group (74.74 ± 2.42%; Figure 5(c)). The percentage of CRX nuclear-positive cells in the JAG1 treatment group (81.42 ± 6.49%, *p* < 0.001) was significantly higher than that in the regular induction group (40.72 ± 7.01%), and the percentage of CRX nuclear-positive cells was also significantly higher in DAPT treatment group (68.94 ± 4.77%, *p* < 0.001). The percentages of TUBB3-positive cells were lower in the JAG1 treatment group (14.23 ± 4.68%, *p* < 0.05), but higher in the DAPT treatment group (28.93 ± 3.40%, *p* < 0.05), compared to that in the regular induction group (21.25 ± 6.18%). In addition, the percentage of POU4F2 nuclear-positive cells was higher in the DAPT treatment group (46.12 ± 5.84%, *p* < 0.01) than that in the regular induction group (35.10 ± 7.86%).

**Figure 6:**
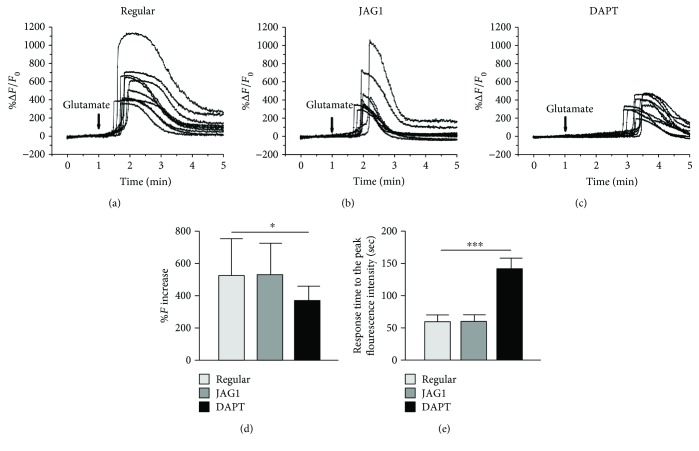
Glutamate-evoked calcium response in retinal-induced human adipose-derived stem cells upon Notch signaling modulation. Calcium transient profiles of retinal-induced ASCs in (a) the regular induction group, (b) JAG1 group, and (c) DAPT group. (d) Histogram showing the fluorescence intensity changes (%Δ*F*/*F*_0_). (e) Histogram showing the response time to the peak fluorescence intensity after the addition of glutamate (^∗^ *p* < 0.05 and ^∗∗^ *p* < 0.01).

Upon glutamate stimulation, the fluorescent intensity changes in the JAG1 treatment group (532.10 ± 191.77%; Figure 6(b)) were similar to those in the regular induction group (525.18 ± 228.80%; Figure 6(a)). On the contrary, the fluorescent intensity changes in the DAPT treatment group (369.36 ± 90.43%; Figures 6(c), 6(d)) were significantly smaller than those in the regular induction group (*p* < 0.05). Besides, the time response to the glutamate stimulation in the DAPT-treated cells was also significantly delayed (141.60 ± 16.24 sec, *p* < 0.001; Figure 6(e)), compared to the regular induction group (59.40 ± 10.29 sec). The JAG1 treatment did not affect the response of the induced ASCs to glutamate stimulation (60.30 ± 9.94 sec).

Collectively, our results suggested that Notch signaling activation further enhanced the expression of retinal markers and produced more RPCs and photoreceptor precursors without affecting the response to glutamate stimulation during the human ASCs' retinal differentiation process. In contrast, Notch signaling inhibition produced more RGCs but suppressed the response to glutamate stimulation.

## 4. Discussion

Stem cells isolated from adipose tissues are predominantly MSCs, and they can differentiate into adipocytes, osteoblasts, and chondrocytes *in vitro* upon induction (Figure 1). In addition to the mesodermal lineage, ASCs have also been reported to differentiate into the ectodermal lineage, including neuronal and retinal lineages, under specific induction conditions [18, 19]. Compared to the teeth, adipose tissues are more conveniently accessible and easily obtained by liposuction with minimal invasiveness. Furthermore, human ASCs can abundantly be isolated with a high proliferative capacity [20]. They also have a low risk of inducing teratoma after transplantation [7]. Human ASCs should be a promising cell source for retinal cell synthesis, *in vitro* disease modeling, and cell replacement therapy.

The noggin/Dkk-1/IGF-1 protocol was originally developed for the retinal differentiation of human ESCs [3]. We have applied this protocol to induce human PDLSCs to the retinal lineage [5, 6]. Noggin and Dkk-1 are the respective inhibitors of the BMP and Wnt signaling pathways. Antagonizing the BMP and Wnt signaling pathways promote neural plate development and retinogenesis [21, 22], whereas the addition of IGF-1 specifies the retinal progenitor identity [3]. In this study, the noggin/Dkk-1/IGF-1 treatment reprogrammed human ASCs with distinct neuron-like morphology (Figure 2(b)). Cell aggregation promotes the induction into the neuronal lineage [23], whereas the Matrigel assists the maturation of neuronal cells [24]. In contrast, human ASCs, without the noggin/Dkk-1/IGF-1 treatment, retained the fibroblast-like morphology and are highly proliferative.

The noggin/Dkk-1/IGF-1 treatment on adult stem cells is specific. Our previous studies showed that, under noggin/Dkk-1/IGF-1 induction, human PDLSCs could express eye field transcription factors (*Pax6*, *Rx*, *Lhx*, and *Otx2*), photoreceptor markers (*Nrl*, *Rhodopsin*, and its kinase), and neuronal and retinal ganglion cell markers (*ATOH7*, *POU4F2*, *β-III tubulin*, *MAP 2*, *TAU*, *NEUROD1*, and *SIX3*). Moreover, the induced PDLSCs showed a significant response to excitatory glutamate [5, 6]. In this study, RPC (*PAX6* and *NES*), photoreceptor (*CRX*, *NRL*, *RHO*, and *RCVRN*), and RGC (*ATOH7* and *TUBB3*) markers were significantly upregulated during the induction treatment period in a time-dependent manner (Figure 3). In addition, as the inner retinal neurons possess the glutamate receptors [25, 26], the neurotransmission function of the retinal-induced ASCs was evaluated by the glutamate-induced calcium response (Figure 4). Similar to our previous study on human PDLSCs, a robust increase in fluorescence intensities was observed in the retinal-induced ASCs after glutamate stimulation, confirming that the retinal-induced ASCs possess an excitable membrane property, like the developing retinal neurons [26, 27]. Collectively, our results suggested that human ASCs can transdifferentiate into functional retinal cells under the noggin/Dkk-1/IGF-1 induction treatment. The noggin/Dkk-1/IGF-1 induction treatment is a chemical-based strategy with a precisely defined formula and condition for retinal differentiation, which can be adopted to synthesize the retinal cells for clinical applications.

Notch signaling is an evolutionarily conserved mechanism that is used by metazoans to control cell fates through local cell interactions [11]. In vertebrates, there are four Notch receptors (Notch-1, -2, -3, and -4) and two ligands (delta and jagged). The activation of Notch signaling induces the expression of transcriptional repressor genes, such as *HES1*, which represses the proneural gene expression and thereby inhibits neuronal differentiation. Thus, the activation of Notch signaling leads to the maintenance of the neural stem cell population, whereas the inactivation of Notch signaling induces neuronal differentiation and depletes the neural stem cell population [28, 29]. Downregulation of Notch-1 promotes neuronal differentiation of bone marrow-derived MSCs [30]. However, inactivation of Notch signaling leads to the complete loss of neural stem cells in both the developing and adult telencephalon, indicating an absolute requirement of Notch signaling for the maintenance of neural stem cells and a proper control of neurogenesis [29, 31].

In this study, we confirmed the efficacy of Notch signaling modulation with the upregulation of *HES1* in JAG1 treatment (as the Notch signaling activator) and its downregulation in DAPT treatment (as the Notch signaling inhibitor; Figure 5(a)). Notably, *NOTCH1* expression is also upregulated in the JAG1 treatment group, indicating that a positive feedback loop was established in human ASCs upon Notch signaling activation during the retinal differentiation process. Notch signaling activation by JAG1, on top of the regular noggin/Dkk-1/IGF-1 induction treatment, further enhances the expression of retinal progenitor and precursor markers (*PAX6*, *NES*, *CRX*, *NRL*, and *ATOH7*) as well as mature photoreceptor (*RHO*) and RGC (*TUBB3*) markers (Figures 5(a) and 5(b)). JAG1 treatment also produced more PAX6- and CRX-positive cells, but less POU4F2- and TUBB3-positive cells (Figure 5(c)), indicating that Notch signaling activation can enhance the maintenance or production of retinal progenitor and precursor markers during human adult stem cell retinal differentiation. Similar to previous reports on the retinal development of zebrafish, xenopus, chick, and mice, the role of Notch signaling is to preserve a pool of undifferentiated progenitor cells, acquire stem cell properties, and prevent differentiation [14–16, 32, 33]. In contrast, Notch signaling inhibition by DAPT downregulated the expression of the *NES* gene, but showed more POU4F2- and TUBB3-positive cells, suggesting that Notch signaling inhibition would reduce the RPC population but increase the production towards the retinal ganglion lineage. Yet, DAPT treatment also delayed the response of glutamate stimulation in the retinal-induced ASCs (Figure 6), suggesting that Notch signaling could be involved in the glutamate signaling transmission in retinal-induced ASCs. Apart from its direct regulation in the differentiation processes, Notch signaling could also interact with other signaling pathways, including the BMP and Wnt signaling pathways [34, 35], and the cell fate determination would be multifactorial [11]. Notch signaling promotes cyclic expression of both repressors and activators of proneural fate specification in progenitor cells [36, 37], indicating that the role of Notch signaling in progenitor cells is not static, but dynamic or oscillatory. Besides, the prolonged activation of Notch signaling would not interfere in the normal progression of progenitor temporal states [38], and a continuous supply of RPCs is required for the production of differentiated neurons and completes retinal development [39]. The molecular mechanisms of Notch signaling modulation in adult stem cell retinal differentiation and the optimal conditions of ASCs for retinal differentiation, as well as the neuroprotective effects of ASCs, require further investigations.

## 5. Conclusion

In summary, human ASCs possess the retinal differentiation potential upon noggin/Dkk-1/IGF-1 induction treatment, and the synthesis of retinal progenitor and precursor cells can further be enhanced by Notch signaling activation. Human ASCs should be a promising autologous source of adult stem cells for retinal cell synthesis, *in vitro* disease modeling, and stem cell replacement therapy in the future.

## Data Availability

The data used to support the findings of this study are available from the corresponding author upon request.

## Supplementary Materials

Supplementary 1 Supplementary Table 1: antibodies used in flow cytometry, immunofluorescence, and immunoblotting analyses.

Supplementary 2 Supplementary Table 2: primers for gene expression analysis.

Supplementary 3 Supplementary Figure 1: assessment of lipid deposition, alkaline phosphatase, and calcium deposition in retinal-induced ASCs.

Supplementary 4 Supplemental Video 1: glutamate-evoked calcium response in the induction group.

Supplementary 5 Supplemental Video 2: glutamate-evoked calcium response in the negative control group.
