# Supplementary material for: Notch Signaling Activation Enhances Human Adipose-Derived Stem Cell Retinal Differentiation
Source: Stem Cells Int. 2018 Oct 16;2018:9201374. doi: 10.1155/2018/9201374 (PMC6206515; doi:10.1155/2018/9201374)
Supplement: Supplementary 1 — Supplementary Table 1: antibodies used in flow cytometry, immunofluorescence, and immunoblotting analyses. [file 9201374.f1.docx]

**Supplementary table 1. Antibodies used in flow cytometry, immunofluorescence and immunoblotting analyses**

| **Antigen** | **Antibodies Conjugated with** | **Supplier** | **Catalog No.** | **Source** | **Dilution factor** |
| --- | --- | --- | --- | --- | --- |
| CD44 | PE | BD | PMG 561858 | Mouse | 1:5 |
| CD90 | APC | BD | PMG 561971 | Mouse | 1:20 |
| CD73 | PerCP-Cy5.5 | BD | PMG 561260 | Mouse | 1:20 |
| CD105 | FITC | BD | PMG 561443 | Mouse | 1:20 |
| CD14 | PE | BD | PMG 561707 | Mouse | 1:5 |
| CD34 | APC | BD | PMG 560940 | Mouse | 1:5 |
| CD45 | FITC | BD | PMG 560976 | Mouse | 1:20 |
| isotype | PE | BD | PMG 555743 | Mouse | 1:5 |
| isotype | APC | BD | PMG 555751 | Mouse | 1:5 |
| isotype | FITC | BD | PMG 555748 | Mouse | 1:5 |
| isotype | PerCP-Cy5.5 | BD | PMG 550795 | Mouse | 1:20 |
| PAX6 |  | Abcam | ab195045 | Rabbit | 1:100^†^ or 1:1000^‡^ |
| CRX |  | Novus | H00001406-M02 | Mouse | 1:100^†^ or 1:200^‡^ |
| RHO |  | Thermo | MA1-722 | Mouse | 1:100^†^ or 1:1000^‡^ |
| POU4F2 |  | Abcam | ab56026 | Rabbit | 1:100^†^ or 1:1000^‡^ |
| TUBB3 |  | CST | CST 5568S | Rabbit | 1:100^†^ or 1:1000^‡^ |

^†^ represents the dilution factor used in immunofluorescence analysis; ^‡^ represents the dilution factor used in immunoblotting analysis
