# Supplementary material for: Notch Signaling Activation Enhances Human Adipose-Derived Stem Cell Retinal Differentiation
Source: Stem Cells Int. 2018 Oct 16;2018:9201374. doi: 10.1155/2018/9201374 (PMC6206515; doi:10.1155/2018/9201374)
Supplement: Supplementary 2 — Supplementary Table 2: primers for gene expression analysis. [file 9201374.f2.docx]

**Supplementary table 2: Primers for gene expression analysis**

| **Gene** |  | **Sequence (5' > 3')** | **Tm (^o^C)** | **Product size (bp)** | **Accession No.** |
| --- | --- | --- | --- | --- | --- |
| *AX6* | F：AGAGGTCAGGCTTCGCTAATG  R：TCAGATTCCTATGCTGATTGGTGA | | 60 | 110 | NM_000280.4 |
| *CRX* | F：AGGGTTCAGGTTTGGTTCAAG  R：GAGGGGGACTGTAGGAATCTG | | 60 | 193 | NM_000554.4 |
| *NRL* | F：CATTGGGGCTGAGTCCTGAAGA  R：TTTAGCTCCCGCACAGACATCG | | 60 | 178 | NM_006177.3 |
| *ATOH7* | F：CAGACCTATGGACGCAATCA  R：TTTTCACAGCAATCAACCCA | | 60 | 105 | NM_145178.3 |
| *POU4F2* | F：TCCAACCCCACCGAGCAATA  R：TCTGGGAGACGATGTCCACG | | 60 | 92 | NM_004575.2 |
| *RHO* | F：CGGCTGGTCCAGGTACATC  R：GTGAAGACGAGCTGCCCATA | | 60 | 168 | NM_000539.3 |
| *RCVRN* | F：GGAGATCGTCATGGCTATTTTCAA  R：GCCAGTGTCCCCTCAATGAA | | 60 | 165 | NM_002903.2 |
| *NES* | F：GATCGCTCAGGTCCTGGAAG  R：CTTGGGGTCCTGAAAGCTGA | | 60 | 160 | NM_006617.1 |
| *TUBB3* | F：GGGAGATCGTGCACATCCAG  R：GAGGCACGTACTTGTGAGAAGA | | 60 | 180 | NM_006086.3 |
| *NOTCH1* | F：ATGCAGAACAACAGGGAGGAG  R：CACCAGGTTGTACTCGTCCAG | | 60 | 192 | NM_017617.4 |
| *HES1* | F：TCAACACGACACCGGATAAAC  R：GCCGCGAGCTATCTTTCTTCA | | 60 | 153 | NM_005524.3 |
| *ACTB* | F：CCCTGGACTTCGAGCAAGAG  R：ACTCCATGCCCAGGAAGGAA | | 60 | 153 | NM_001101.3 |
