# Supplementary material for: Notch Signaling Activation Enhances Human Adipose-Derived Stem Cell Retinal Differentiation
Source: Stem Cells Int. 2018 Oct 16;2018:9201374. doi: 10.1155/2018/9201374 (PMC6206515; doi:10.1155/2018/9201374)
Supplement: Supplementary 3 — Supplementary Figure 1: assessment of lipid deposition, alkaline phosphatase, and calcium deposition in retinal-induced ASCs. [file 9201374.f3.pdf]

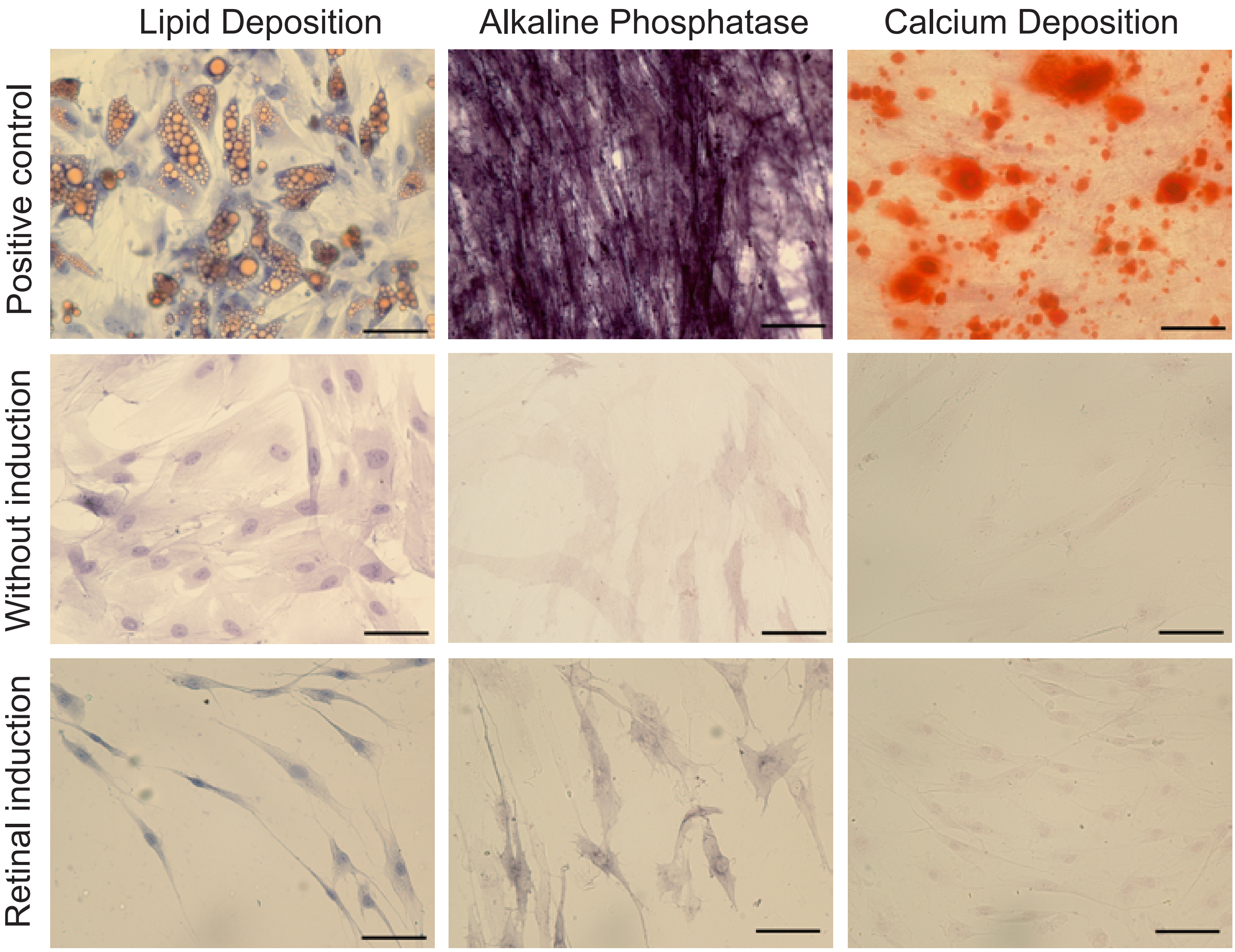
Lipid Deposition
Alkaline Phosphatase
Calcium Deposition
Positive control
Without induction
Retinal induction
